# *cis*-Dichloridobis(2-isocyano­phenyl 4-meth­oxy­benzoate)palladium(II) chloro­form monosolvate

**DOI:** 10.1107/S1600536812045801

**Published:** 2012-11-14

**Authors:** Alexander Tskhovrebov, Matti Haukka

**Affiliations:** a Department of Chemistry, Saint-Petersburg State University, Universitetsky Pr. 26, 198504 Stary Petergof, Russian Federation; b University of Jyväskylä, Department of Chemistry, PO Box 35, 40014 University of Jyväskylä, Finland

## Abstract

In the title compound, [PdCl_2_(C_15_H_11_NO_3_)_2_]·CHCl_3_, the Pd^II^ atom adopts a slightly distorted square-planar coordination geometry composed of two Cl atoms in *cis* positions and two C atoms from isocyano­phenyl ligands. The mol­ecular conformation is stabilized by π–π stacking inter­actions [shortest centroid–centroid distance = 3.600 (1) Å] between substituted benzene rings of different ligands. The crystal packing is characterized by C—H⋯O and C—H⋯Cl inter­actions involving the chloro­form solvent mol­ecules.

## Related literature

For further information on acyclic diamino­carbenes, see: Slaughter (2012 ▶); Boyarskiy *et al.* (2012 ▶). For background to the Passerini reaction, see: Banfi & Riva (2005 ▶). For novel metal-mediated coupling as a route to cyclic carbenes and amino­carbene complexes, see: Luzyanin *et al.* (2009*a* ▶,*b* ▶); Tskhovrebov *et al.* (2011 ▶); Chay *et al.* (2012 ▶). For related structures, see: Davies *et al.* (1996 ▶); Bertani *et al.* (1991 ▶); Bonati & Minghetti (1970 ▶); Luzyanin *et al.* (2009*a* ▶,*b* ▶); Michelin *et al.* (1988*a* ▶,*b* ▶); Rourke (2007 ▶). For bond lengths in coordin­ation complexes, see: Orpen *et al.* (1989 ▶).
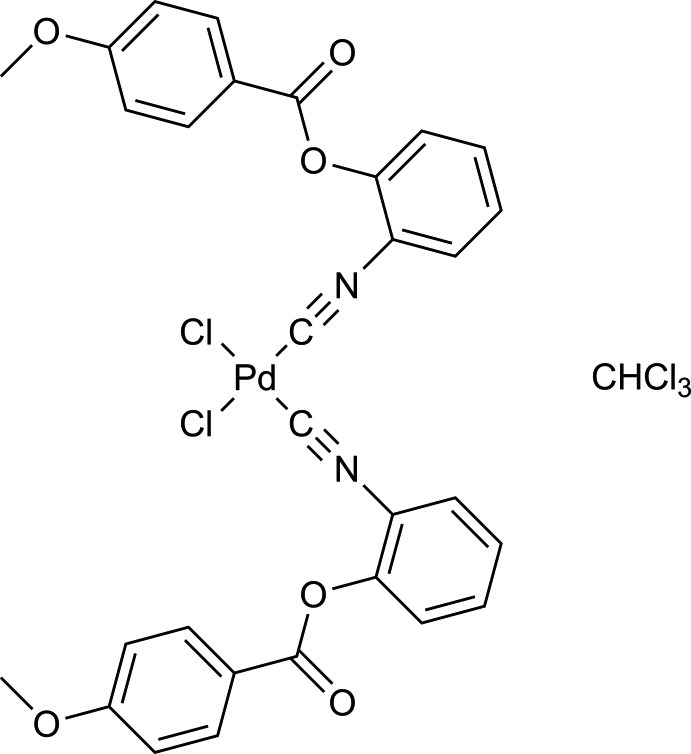


## Experimental

### 

#### Crystal data

[PdCl_2_(C_15_H_11_NO_3_)_2_]·CHCl_3_
*M* *_r_* = 803.16
Orthorhombic, 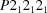

*a* = 7.4457 (1) Å
*b* = 12.1352 (4) Å
*c* = 36.1109 (11) Å
*V* = 3262.80 (15) Å^3^
*Z* = 4
Mo *K*α radiation
μ = 1.02 mm^−1^
*T* = 100 K
0.35 × 0.23 × 0.10 mm

#### Data collection

Nonius KappaCCD diffractometer
Absorption correction: multi-scan (*SADABS*; Sheldrick, 2008 ▶)*T*_min_ = 0.717, *T*_max_ = 0.903
24908 measured reflections
9228 independent reflections
7397 reflections with *I* > 2σ(*I*)
*R*_int_ = 0.046

#### Refinement

*R*[*F* ^2^ > 2σ(*F* ^2^)] = 0.037
*wR*(*F* ^2^) = 0.067
*S* = 1.01
9228 reflections
408 parameters
H-atom parameters constrained
Δρ_max_ = 0.61 e Å^−3^
Δρ_min_ = −0.79 e Å^−3^
Absolute structure: Flack (1983 ▶), 3936 Friedel pairs
Flack parameter: −0.011 (17)

### 

Data collection: *COLLECT* (Nonius, 1997 ▶); cell refinement: *DENZO* and *SCALEPACK* (Otwinowski & Minor, 1997 ▶); data reduction: *DENZO* and *SCALEPACK*; program(s) used to solve structure: *SHELXS97* (Sheldrick, 2008 ▶); program(s) used to refine structure: *SHELXL97* (Sheldrick, 2008 ▶); molecular graphics: *DIAMOND* (Brandenburg, 2009 ▶); software used to prepare material for publication: *SHELXL97*.

## Supplementary Material

Crystal structure: contains datablock(s) I, global. DOI: 10.1107/S1600536812045801/zq2184sup1.cif

Structure factors: contains datablock(s) I. DOI: 10.1107/S1600536812045801/zq2184Isup2.hkl

Additional supplementary materials:  crystallographic information; 3D view; checkCIF report

## Figures and Tables

**Table 1:** Selected bond lengths (Å)

Pd1—C16	1.935 (3)
Pd1—C1	1.947 (3)
Pd1—Cl2	2.2979 (7)
Pd1—Cl1	2.2994 (7)

**Table 2:** Hydrogen-bond geometry (Å, °)

*D*—H⋯*A*	*D*—H	H⋯*A*	*D*⋯*A*	*D*—H⋯*A*
C4—H4⋯O2^i^	0.95	2.53	3.193 (4)	127
C6—H6⋯O6^ii^	0.95	2.53	3.433 (4)	158
C19—H19⋯O5^iii^	0.95	2.37	3.182 (3)	143
C20—H20⋯Cl1^iv^	0.95	2.80	3.622 (3)	145
C31—H31⋯Cl1^v^	1.00	2.77	3.607 (3)	141
C31—H31⋯Cl2^v^	1.00	2.67	3.513 (3)	142

Symmetry codes: (i) 
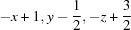
; (ii) 
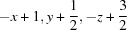
; (iii) 
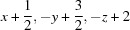
; (iv) 
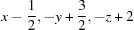
; (v) 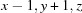.

**Fig. 1.**
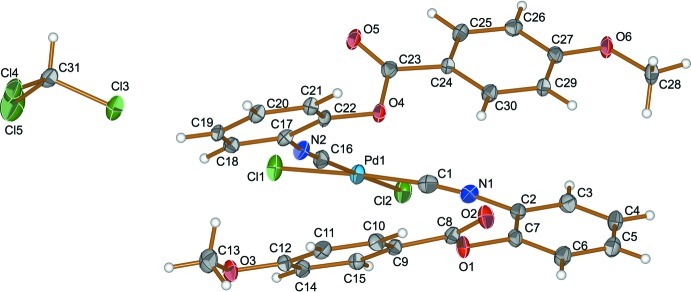
The molecular structure of the title compound, with displacement ellipsoids drawn at the 50% probability level. H atoms are presented as small spheres of arbitrary radius.

## supplementary crystallographic information

### Comment

Isocyanides are important organic reagents used in multicomponent reactions such as, *e.g.*, Ugi and Passerini reactions (Banfi & Riva, 2005). Metal complexes of isocyanides could be used as precursors for the generation of coordinated N-heterocyclic carbenes (NHC's) and acyclic diaminocarbenes (ADS's) (Slaughter, 2012). In turn, Pd^II^-NHC and Pd^II^-ADC systems are particularly interesting since they are used as catalysts in a wide range of cross-coupling reactions (Boyarskiy *et al.*, 2012). Recently, it was observed that the coupling of Pd^II^-bound isocyanides and various nucleophiles leads to the formation of cyclic carbenes (Luzyanin *et al.*, 2009*b*) and ADC complexes (Luzyanin *et al.*, 2009*a*; Tskhovrebov *et al.*, 2011; Chay *et al.*, 2012), which could not be obtained by the common methods for the generation of metal carbenes. Here we report the structure of a new isocyanide complex that could be used as a starting material for generation of various palladium carbenes.

In the title compound, the isocyanide ligands are mutually in the *cis*-position (Fig. 1) insofar as the ligated RNC species exhibit higher *trans*-effect than the chlorides (Davies *et al.*, 1996). The fragments C–N–C–Pd in both complexes are almost linear, *viz*., the angles N1–C1–Pd1 and N2–C16–Pd1 are 174.2 (2)° and 177.4 (3)°, respectively. The angles C2–N1–C1 and C17–N2–C16 are 174.3 (3)° and 172.0 (3)°, correspondingly. In the isocyanide moieties, the C≡N triple bonds [C1–N1 1.141 (3) Å and C16–N2 1.150 (3) Å] are close to those in some other palladium-isocyanide complexes (Bertani *et al.*, 1991; Bonati & Minghetti, 1970; Luzyanin *et al.*, 2009*a*,*b*; Michelin *et al.*, 1988*a*,*b*; Orpen *et al.*, 1989; Rourke, 2007). The molecular conformation is stabilized by π-π stacking interactions [shortest centroid-centroid distance = 3.600 (1) Å] between the substituted benzene rings C9–C15 and C17–C22 of different ligands. The crystal packing is characterized by intermolecular C-H···O and C-H···Cl interactions involving the chloroform solvent molecules (Table 1).

### Experimental

The title compound was synthesized by the addition of 2 equiv of 2-isocyanophenyl-4-methoxybenzoate into a chloroform solution of [PdCl_2_(MeCN)_2_]. The solid product was dissolved and recrystallized by slow evaporation from a solution of Et_2_O/CHCl_3_ (1:1, *v*/*v*).

### Refinement

All H atoms were positioned geometrically and constrained to ride on their parent atoms, with C—H = 0.95 Å and *U*_iso_ = 1.2*U*_eq_(C) for aromatic H atoms, with C—H = 1.00 Å and *U*_iso_ = 1.2*U*_eq_(C) for methine H atoms, and with C—H = 0.98 Å and *U*_iso_ = 1.5*U*_eq_(C) for methyl H atoms. The highest peak is located 1.28 Å from atom Cl6 and the deepest hole is located 0.78 Å from atom Pd1.

### Crystal data

**Table:**

[PdCl_2_(C_15_H_11_NO_3_)_2_]·CHCl_3_	*F*(000) = 1608
*M**_r_* = 803.16	*D*_x_ = 1.635 Mg m^−^^3^
Orthorhombic, *P*2_1_2_1_2_1_	Mo *K*α radiation, λ = 0.71073 Å
Hall symbol: P 2ac 2ab	Cell parameters from 24908 reflections
*a* = 7.4457 (1) Å	θ = 3.2–30.0°
*b* = 12.1352 (4) Å	µ = 1.02 mm^−^^1^
*c* = 36.1109 (11) Å	*T* = 100 K
*V* = 3262.80 (15) Å^3^	Block, colourless
*Z* = 4	0.35 × 0.23 × 0.10 mm

### Data collection

**Table:**

Nonius KappaCCD diffractometer	9228 independent reflections
Radiation source: fine-focus sealed tube	7397 reflections with *I* > 2σ(*I*)
Horizontally mounted graphite crystal monochromator	*R*_int_ = 0.046
Detector resolution: 9 pixels mm^-1^	θ_max_ = 30.0°, θ_min_ = 3.2°
φ scans and ω scans with κ offset	*h* = −10→10
Absorption correction: multi-scan (*SADABS*; Sheldrick, 2008)	*k* = −17→15
*T*_min_ = 0.717, *T*_max_ = 0.903	*l* = −50→41
24908 measured reflections	

### Refinement

**Table:**

Refinement on *F*^2^	Secondary atom site location: difference Fourier map
Least-squares matrix: full	Hydrogen site location: inferred from neighbouring sites
*R*[*F*^2^ > 2σ(*F*^2^)] = 0.037	H-atom parameters constrained
*wR*(*F*^2^) = 0.067	*w* = 1/[σ^2^(*F*_o_^2^) + (0.0262*P*)^2^] where *P* = (*F*_o_^2^ + 2*F*_c_^2^)/3
*S* = 1.01	(Δ/σ)_max_ = 0.004
9228 reflections	Δρ_max_ = 0.61 e Å^−^^3^
408 parameters	Δρ_min_ = −0.79 e Å^−^^3^
0 restraints	Absolute structure: Flack (1983), 3936 Friedel pairs
Primary atom site location: structure-invariant direct methods	Flack parameter: −0.011 (17)

### Special details

**Table:**

Geometry. All e.s.d.'s (except the e.s.d. in the dihedral angle between two l.s. planes) are estimated using the full covariance matrix. The cell e.s.d.'s are taken into account individually in the estimation of e.s.d.'s in distances, angles and torsion angles; correlations between e.s.d.'s in cell parameters are only used when they are defined by crystal symmetry. An approximate (isotropic) treatment of cell e.s.d.'s is used for estimating e.s.d.'s involving l.s. planes.
Refinement. Refinement of *F*^2^ against ALL reflections. The weighted *R*-factor *wR* and goodness of fit *S* are based on *F*^2^, conventional *R*-factors *R* are based on *F*, with *F* set to zero for negative *F*^2^. The threshold expression of *F*^2^ > σ(*F*^2^) is used only for calculating *R*-factors(gt) *etc*. and is not relevant to the choice of reflections for refinement. *R*-factors based on *F*^2^ are statistically about twice as large as those based on *F*, and *R*- factors based on ALL data will be even larger.

### Fractional atomic coordinates and isotropic or equivalent isotropic displacement parameters (Å^2^)

**Table:**

	*x*	*y*	*z*	*U*_iso_*/*U*_eq_	
Pd1	0.70069 (3)	0.468290 (17)	0.888077 (5)	0.01742 (5)	
Cl1	0.77179 (10)	0.36814 (6)	0.940105 (18)	0.02588 (17)	
Cl2	0.78655 (11)	0.32749 (5)	0.849385 (18)	0.02437 (15)	
Cl3	0.19677 (12)	1.19482 (6)	0.947449 (18)	0.02811 (16)	
Cl4	0.16546 (10)	1.10483 (7)	0.873361 (19)	0.03021 (18)	
Cl5	0.30198 (14)	1.32462 (7)	0.88439 (2)	0.0512 (2)	
O1	0.7349 (2)	0.81404 (15)	0.82187 (5)	0.0201 (4)	
O2	0.5584 (2)	0.96590 (18)	0.81736 (5)	0.0243 (4)	
O3	0.8859 (2)	0.99125 (16)	0.98090 (5)	0.0236 (5)	
O4	0.3493 (2)	0.79408 (15)	0.89629 (5)	0.0176 (4)	
O5	0.1775 (3)	0.65489 (16)	0.91802 (5)	0.0243 (5)	
O6	0.0757 (3)	0.58547 (17)	0.74567 (5)	0.0231 (5)	
N1	0.6421 (3)	0.60184 (19)	0.81643 (6)	0.0197 (5)	
N2	0.5755 (3)	0.66170 (19)	0.93709 (6)	0.0181 (5)	
C1	0.6542 (3)	0.5533 (2)	0.84336 (8)	0.0202 (6)	
C2	0.6443 (3)	0.6656 (2)	0.78406 (7)	0.0181 (6)	
C3	0.6047 (4)	0.6183 (3)	0.75010 (7)	0.0213 (6)	
H3	0.5760	0.5422	0.7484	0.026*	
C4	0.6076 (4)	0.6834 (3)	0.71872 (8)	0.0242 (7)	
H4	0.5802	0.6522	0.6953	0.029*	
C5	0.6502 (4)	0.7939 (3)	0.72142 (8)	0.0246 (7)	
H5	0.6505	0.8383	0.6997	0.030*	
C6	0.6925 (4)	0.8410 (2)	0.75529 (7)	0.0220 (6)	
H6	0.7245	0.9166	0.7567	0.026*	
C7	0.6877 (4)	0.7772 (2)	0.78685 (7)	0.0175 (6)	
C8	0.6627 (4)	0.9124 (2)	0.83496 (7)	0.0175 (6)	
C9	0.7281 (3)	0.9349 (2)	0.87254 (7)	0.0158 (6)	
C10	0.6814 (3)	1.0340 (2)	0.88931 (7)	0.0187 (5)	
H10	0.6137	1.0867	0.8758	0.022*	
C11	0.7323 (3)	1.0570 (2)	0.92547 (7)	0.0186 (6)	
H11	0.7003	1.1252	0.9366	0.022*	
C12	0.8307 (3)	0.9794 (2)	0.94535 (7)	0.0185 (6)	
C13	0.8259 (4)	1.0869 (2)	1.00095 (7)	0.0293 (7)	
H13A	0.8686	1.1536	0.9884	0.044*	
H13B	0.8739	1.0847	1.0262	0.044*	
H13C	0.6944	1.0875	1.0019	0.044*	
C14	0.8794 (3)	0.8798 (2)	0.92857 (7)	0.0176 (6)	
H14	0.9465	0.8267	0.9421	0.021*	
C15	0.8303 (3)	0.8586 (2)	0.89253 (7)	0.0173 (6)	
H15	0.8662	0.7916	0.8811	0.021*	
C16	0.6242 (4)	0.5885 (2)	0.91953 (7)	0.0194 (6)	
C17	0.5048 (4)	0.7558 (2)	0.95387 (7)	0.0165 (6)	
C18	0.5512 (3)	0.7851 (2)	0.98970 (7)	0.0176 (6)	
H18	0.6306	0.7403	1.0038	0.021*	
C19	0.4803 (3)	0.8805 (2)	1.00472 (7)	0.0173 (6)	
H19	0.5105	0.9015	1.0293	0.021*	
C20	0.3652 (3)	0.9456 (2)	0.98394 (7)	0.0176 (6)	
H20	0.3170	1.0110	0.9944	0.021*	
C21	0.3197 (4)	0.9165 (2)	0.94812 (7)	0.0171 (6)	
H21	0.2407	0.9617	0.9341	0.021*	
C22	0.3893 (3)	0.8223 (2)	0.93303 (7)	0.0152 (6)	
C23	0.2352 (3)	0.7055 (2)	0.89214 (8)	0.0182 (6)	
C24	0.1980 (4)	0.6801 (2)	0.85274 (7)	0.0153 (5)	
C25	0.1211 (4)	0.5787 (2)	0.84476 (7)	0.0198 (6)	
H25	0.0940	0.5293	0.8644	0.024*	
C26	0.0834 (3)	0.5485 (2)	0.80881 (7)	0.0203 (6)	
H26	0.0349	0.4778	0.8035	0.024*	
C27	0.1174 (3)	0.6232 (2)	0.78018 (7)	0.0185 (6)	
C28	0.1047 (4)	0.6582 (3)	0.71486 (7)	0.0269 (7)	
H28A	0.0354	0.7260	0.7185	0.040*	
H28B	0.0658	0.6221	0.6920	0.040*	
H28C	0.2327	0.6763	0.7131	0.040*	
C29	0.1902 (4)	0.7258 (2)	0.78777 (7)	0.0185 (6)	
H29	0.2117	0.7765	0.7682	0.022*	
C30	0.2316 (3)	0.7546 (2)	0.82398 (7)	0.0182 (6)	
H30	0.2825	0.8247	0.8292	0.022*	
C31	0.1513 (4)	1.2255 (2)	0.90068 (7)	0.0237 (7)	
H31	0.0267	1.2557	0.8988	0.028*	

### Atomic displacement parameters (Å^2^)

**Table:**

	*U*^11^	*U*^22^	*U*^33^	*U*^12^	*U*^13^	*U*^23^
Pd1	0.02268 (10)	0.01504 (10)	0.01454 (9)	0.00299 (10)	0.00058 (9)	−0.00068 (9)
Cl1	0.0409 (4)	0.0209 (4)	0.0159 (3)	0.0074 (3)	−0.0020 (3)	−0.0008 (3)
Cl2	0.0353 (4)	0.0195 (3)	0.0184 (3)	0.0051 (4)	0.0003 (3)	−0.0039 (3)
Cl3	0.0448 (4)	0.0220 (4)	0.0175 (3)	0.0023 (4)	−0.0028 (4)	0.0009 (3)
Cl4	0.0401 (4)	0.0340 (4)	0.0165 (3)	0.0044 (4)	−0.0049 (3)	−0.0018 (3)
Cl5	0.0628 (5)	0.0512 (6)	0.0397 (5)	−0.0301 (5)	−0.0031 (6)	0.0165 (4)
O1	0.0291 (11)	0.0193 (10)	0.0119 (9)	0.0024 (9)	−0.0041 (8)	−0.0030 (8)
O2	0.0310 (11)	0.0232 (11)	0.0186 (11)	0.0062 (11)	−0.0058 (8)	0.0005 (10)
O3	0.0283 (10)	0.0287 (13)	0.0139 (10)	0.0011 (9)	−0.0028 (8)	−0.0068 (9)
O4	0.0247 (10)	0.0177 (10)	0.0104 (10)	−0.0043 (8)	−0.0010 (7)	0.0006 (7)
O5	0.0324 (11)	0.0296 (12)	0.0109 (9)	−0.0083 (10)	0.0024 (9)	−0.0012 (8)
O6	0.0312 (11)	0.0284 (12)	0.0097 (10)	−0.0021 (10)	−0.0025 (8)	−0.0009 (9)
N1	0.0214 (12)	0.0170 (13)	0.0207 (13)	0.0035 (10)	0.0028 (10)	0.0013 (10)
N2	0.0240 (12)	0.0146 (13)	0.0157 (12)	0.0036 (10)	0.0030 (10)	0.0016 (10)
C1	0.0197 (14)	0.0167 (16)	0.0240 (15)	0.0023 (11)	0.0033 (11)	−0.0053 (12)
C2	0.0170 (13)	0.0228 (16)	0.0145 (14)	0.0005 (12)	0.0000 (11)	0.0000 (12)
C3	0.0226 (14)	0.0219 (16)	0.0194 (15)	−0.0021 (13)	0.0001 (12)	−0.0042 (13)
C4	0.0263 (15)	0.0324 (19)	0.0138 (15)	−0.0011 (14)	0.0004 (12)	−0.0041 (13)
C5	0.0286 (16)	0.0288 (17)	0.0165 (15)	−0.0035 (14)	0.0008 (12)	0.0008 (13)
C6	0.0283 (14)	0.0202 (15)	0.0176 (14)	−0.0023 (15)	0.0017 (14)	−0.0017 (11)
C7	0.0188 (13)	0.0208 (14)	0.0128 (12)	0.0011 (13)	−0.0010 (12)	−0.0037 (11)
C8	0.0210 (15)	0.0163 (14)	0.0150 (13)	−0.0017 (12)	0.0016 (11)	0.0010 (11)
C9	0.0185 (13)	0.0153 (13)	0.0137 (12)	−0.0027 (11)	0.0008 (11)	−0.0002 (10)
C10	0.0196 (12)	0.0157 (12)	0.0207 (13)	0.0019 (13)	−0.0011 (13)	0.0053 (13)
C11	0.0232 (14)	0.0135 (14)	0.0190 (13)	−0.0018 (11)	0.0029 (11)	−0.0015 (10)
C12	0.0182 (13)	0.0235 (15)	0.0138 (13)	−0.0040 (13)	−0.0001 (10)	−0.0022 (12)
C13	0.0363 (18)	0.0322 (18)	0.0192 (15)	−0.0030 (16)	0.0014 (14)	−0.0105 (13)
C14	0.0184 (13)	0.0176 (15)	0.0169 (14)	0.0011 (12)	−0.0009 (11)	0.0029 (12)
C15	0.0167 (13)	0.0175 (14)	0.0177 (14)	−0.0002 (11)	0.0003 (11)	−0.0001 (11)
C16	0.0210 (14)	0.0214 (16)	0.0158 (14)	0.0002 (13)	0.0001 (11)	0.0043 (12)
C17	0.0188 (13)	0.0155 (14)	0.0152 (14)	0.0003 (12)	0.0039 (11)	0.0017 (12)
C18	0.0159 (13)	0.0190 (15)	0.0180 (15)	0.0010 (12)	0.0002 (11)	0.0040 (12)
C19	0.0202 (14)	0.0214 (16)	0.0103 (13)	−0.0052 (13)	0.0006 (11)	−0.0004 (12)
C20	0.0220 (13)	0.0147 (15)	0.0160 (13)	0.0004 (11)	0.0026 (11)	−0.0011 (11)
C21	0.0196 (13)	0.0166 (14)	0.0153 (13)	0.0003 (12)	0.0002 (11)	0.0005 (11)
C22	0.0204 (13)	0.0166 (15)	0.0087 (13)	−0.0034 (12)	0.0002 (10)	0.0009 (11)
C23	0.0189 (13)	0.0194 (14)	0.0162 (14)	0.0025 (11)	0.0020 (11)	0.0006 (12)
C24	0.0158 (12)	0.0183 (13)	0.0118 (12)	−0.0001 (13)	−0.0002 (12)	0.0015 (10)
C25	0.0218 (14)	0.0222 (16)	0.0154 (14)	−0.0002 (12)	0.0018 (11)	0.0032 (12)
C26	0.0214 (13)	0.0196 (17)	0.0198 (14)	−0.0043 (13)	0.0008 (11)	0.0002 (13)
C27	0.0157 (13)	0.0248 (16)	0.0151 (14)	0.0033 (13)	−0.0006 (11)	−0.0011 (12)
C28	0.0300 (16)	0.038 (2)	0.0127 (14)	0.0025 (15)	−0.0032 (13)	0.0035 (13)
C29	0.0196 (13)	0.0231 (15)	0.0128 (12)	0.0003 (14)	−0.0006 (12)	0.0032 (11)
C30	0.0205 (14)	0.0184 (14)	0.0157 (13)	−0.0004 (12)	0.0012 (11)	−0.0024 (11)
C31	0.0267 (15)	0.0252 (17)	0.0192 (15)	0.0005 (13)	−0.0039 (12)	0.0044 (12)

### Geometric parameters (Å, º)

**Table:**

Pd1—C16	1.935 (3)	C10—H10	0.9500
Pd1—C1	1.947 (3)	C11—C12	1.393 (4)
Pd1—Cl2	2.2979 (7)	C11—H11	0.9500
Pd1—Cl1	2.2994 (7)	C12—C14	1.400 (4)
Cl3—C31	1.762 (3)	C13—H13A	0.9800
Cl4—C31	1.768 (3)	C13—H13B	0.9800
Cl5—C31	1.747 (3)	C13—H13C	0.9800
O1—C7	1.387 (3)	C14—C15	1.376 (4)
O1—C8	1.392 (3)	C14—H14	0.9500
O2—C8	1.195 (3)	C15—H15	0.9500
O3—C12	1.356 (3)	C17—C18	1.386 (4)
O3—C13	1.439 (3)	C17—C22	1.400 (4)
O4—C23	1.379 (3)	C18—C19	1.383 (4)
O4—C22	1.402 (3)	C18—H18	0.9500
O5—C23	1.198 (3)	C19—C20	1.386 (4)
O6—C27	1.364 (3)	C19—H19	0.9500
O6—C28	1.437 (3)	C20—C21	1.383 (3)
N1—C1	1.141 (3)	C20—H20	0.9500
N1—C2	1.402 (3)	C21—C22	1.368 (3)
N2—C16	1.150 (3)	C21—H21	0.9500
N2—C17	1.395 (3)	C23—C24	1.482 (3)
C2—C3	1.386 (4)	C24—C25	1.387 (4)
C2—C7	1.396 (4)	C24—C30	1.400 (3)
C3—C4	1.382 (4)	C25—C26	1.378 (3)
C3—H3	0.9500	C25—H25	0.9500
C4—C5	1.381 (4)	C26—C27	1.398 (4)
C4—H4	0.9500	C26—H26	0.9500
C5—C6	1.386 (4)	C27—C29	1.385 (4)
C5—H5	0.9500	C28—H28A	0.9800
C6—C7	1.378 (3)	C28—H28B	0.9800
C6—H6	0.9500	C28—H28C	0.9800
C8—C9	1.467 (3)	C29—C30	1.388 (3)
C9—C10	1.391 (3)	C29—H29	0.9500
C9—C15	1.399 (4)	C30—H30	0.9500
C10—C11	1.388 (3)	C31—H31	1.0000
			
C16—Pd1—C1	92.00 (12)	C14—C15—C9	120.6 (3)
C16—Pd1—Cl2	178.37 (8)	C14—C15—H15	119.7
C1—Pd1—Cl2	86.52 (8)	C9—C15—H15	119.7
C16—Pd1—Cl1	89.24 (8)	N2—C16—Pd1	177.4 (3)
C1—Pd1—Cl1	176.91 (8)	C18—C17—N2	121.4 (2)
Cl2—Pd1—Cl1	92.28 (3)	C18—C17—C22	120.5 (3)
C7—O1—C8	119.2 (2)	N2—C17—C22	118.1 (2)
C12—O3—C13	117.9 (2)	C19—C18—C17	119.1 (3)
C23—O4—C22	115.11 (19)	C19—C18—H18	120.5
C27—O6—C28	117.8 (2)	C17—C18—H18	120.5
C1—N1—C2	174.3 (3)	C18—C19—C20	120.0 (3)
C16—N2—C17	172.0 (3)	C18—C19—H19	120.0
N1—C1—Pd1	174.2 (2)	C20—C19—H19	120.0
C3—C2—C7	121.0 (3)	C21—C20—C19	120.8 (3)
C3—C2—N1	120.5 (3)	C21—C20—H20	119.6
C7—C2—N1	118.5 (2)	C19—C20—H20	119.6
C4—C3—C2	119.1 (3)	C22—C21—C20	119.5 (2)
C4—C3—H3	120.5	C22—C21—H21	120.2
C2—C3—H3	120.5	C20—C21—H21	120.2
C5—C4—C3	120.1 (3)	C21—C22—C17	120.0 (2)
C5—C4—H4	120.0	C21—C22—O4	120.1 (2)
C3—C4—H4	120.0	C17—C22—O4	119.9 (2)
C4—C5—C6	120.9 (3)	O5—C23—O4	122.4 (3)
C4—C5—H5	119.5	O5—C23—C24	125.2 (2)
C6—C5—H5	119.5	O4—C23—C24	112.4 (2)
C7—C6—C5	119.5 (3)	C25—C24—C30	119.5 (2)
C7—C6—H6	120.2	C25—C24—C23	117.4 (2)
C5—C6—H6	120.2	C30—C24—C23	123.0 (2)
C6—C7—O1	124.5 (2)	C26—C25—C24	121.1 (3)
C6—C7—C2	119.4 (2)	C26—C25—H25	119.5
O1—C7—C2	115.9 (2)	C24—C25—H25	119.5
O2—C8—O1	122.4 (2)	C25—C26—C27	119.1 (3)
O2—C8—C9	127.3 (3)	C25—C26—H26	120.4
O1—C8—C9	110.2 (2)	C27—C26—H26	120.4
C10—C9—C15	118.9 (2)	O6—C27—C29	124.9 (2)
C10—C9—C8	118.7 (2)	O6—C27—C26	114.6 (2)
C15—C9—C8	122.3 (2)	C29—C27—C26	120.5 (2)
C11—C10—C9	121.0 (2)	O6—C28—H28A	109.5
C11—C10—H10	119.5	O6—C28—H28B	109.5
C9—C10—H10	119.5	H28A—C28—H28B	109.5
C10—C11—C12	119.5 (2)	O6—C28—H28C	109.5
C10—C11—H11	120.3	H28A—C28—H28C	109.5
C12—C11—H11	120.3	H28B—C28—H28C	109.5
O3—C12—C11	125.1 (2)	C27—C29—C30	120.0 (2)
O3—C12—C14	115.0 (2)	C27—C29—H29	120.0
C11—C12—C14	119.8 (2)	C30—C29—H29	120.0
O3—C13—H13A	109.5	C29—C30—C24	119.8 (3)
O3—C13—H13B	109.5	C29—C30—H30	120.1
H13A—C13—H13B	109.5	C24—C30—H30	120.1
O3—C13—H13C	109.5	Cl5—C31—Cl3	110.17 (15)
H13A—C13—H13C	109.5	Cl5—C31—Cl4	110.14 (15)
H13B—C13—H13C	109.5	Cl3—C31—Cl4	110.39 (16)
C15—C14—C12	120.1 (3)	Cl5—C31—H31	108.7
C15—C14—H14	119.9	Cl3—C31—H31	108.7
C12—C14—H14	119.9	Cl4—C31—H31	108.7

### Hydrogen-bond geometry (Å, º)

**Table:**

*D*—H···*A*	*D*—H	H···*A*	*D*···*A*	*D*—H···*A*
C4—H4···O2^i^	0.95	2.53	3.193 (4)	127
C6—H6···O6^ii^	0.95	2.53	3.433 (4)	158
C19—H19···O5^iii^	0.95	2.37	3.182 (3)	143
C20—H20···Cl1^iv^	0.95	2.80	3.622 (3)	145
C31—H31···Cl1^v^	1.00	2.77	3.607 (3)	141
C31—H31···Cl2^v^	1.00	2.67	3.513 (3)	142

Symmetry codes: (i) −*x*+1, *y*−1/2, −*z*+3/2; (ii) −*x*+1, *y*+1/2, −*z*+3/2; (iii) *x*+1/2, −*y*+3/2, −*z*+2; (iv) *x*−1/2, −*y*+3/2, −*z*+2; (v) *x*−1, *y*+1, *z*.
